# Medical associations and expert committees urge that ethanol be approved as a virucidal active substance for use in hand antiseptics under the European Biocidal Products Regulation, without a CMR classification

**DOI:** 10.3205/dgkh000495

**Published:** 2024-08-21

**Authors:** Axel Kramer, Didier Pittet, Martin Exner, Constanze Wendt

**Affiliations:** 1 Institute of Hygiene and Environmental Medicine, University Medicine Greifswald, Germany; 2 Infection Control Programme, Faculty of Medicine, University of Geneva, Geneva, Switzerland; 3 Institute for Hygiene and Public Health, University Clinics Bonn, Bonn, Germany; VAH – Association for Applied Hygiene c/o Institute for Hygiene and Public Health, Bonn, Germany; 4 MVZ Labor Dr. Limbach, Department of Hygiene, Heidelberg, Germany

**Keywords:** ethanol-based handrubs, transdermal absorption, toxicological harmlessness, biocid classifying, no carcinogenic, no mutagenic, no fetotoxic, virucidal efficacy

## Abstract

**Introduction:**

Since 2007, the classification of ethanol under the Biocidal Products Regulation has paradoxically remained unresolved due to conflicting views among experts and authorities. Initially, there was a discussion about classifying ethanol as carcinogenic. The current proposal to extend its harmonized classification includes, among other things, categorizing it as reproductive toxicity category 2 (“suspected to have CMR potential for humans”; carcinogenic, mutagenic, reprotoxic). If ethanol were classified under reproductive toxicity category 2, it would mean that the only active ingredient in hand antiseptics effective against non-enveloped viruses would no longer be available.

**Scientific assessment of the safety of ethanol-based hand rubs (EBHR):**

Available epidemiological studies do not confirm an increased risk for cancer from EBHR in exposed individuals, except under uncommon or unlikely routes or levels of exposure.

The evidence for ethanol's reprotoxic effect originates from the consumption of alcoholic beverages by pregnant women, where ethanol uptake is incomparably higher. The amount of transdermal ethanol absorption during hand antisepsis is up to ten times lower than the oral intake of beverages containing hidden ethanol, such as apple juice, kefir, or non-alcoholic beer. Blood alcohol levels after using EBHR remain within the physiological range associated with food intake.

**Conclusion:**

There is no epidemiological evidence of toxicity for workers handling ethanol-containing products in industry or using EBHR in healthcare settings. Given that the classification of EBHR as reproductive toxicity category 2 is not supported by current scientific research and that no alternative biocidal active substance in hand rubs is effective against non-enveloped viruses, medical associations and expert committees from Europe, the USA, Canada, the Asia-Pacific region, and the World Society for Virology unequivocally recommend, with the highest priority, that EBHR be approved as an active substance for PT1 biocides and not be classified as a reproductive toxicant in category 2.

## Zusammenfassung

**Einleitung:**

Seit 2007 ist die Einstufung von Ethanol im Rahmen der Biozid-Verordnung paradoxerweise aufgrund widersprüchlicher Ansichten von Experten und Behörden ungelöst geblieben. Ursprünglich war diskutiert worden, Ethanol als krebserregend einzustufen. Die derzeitige Absicht, die harmonisierte Einstufung zu erweitern, sieht u.a. eine Einstufung als Reproduktionstoxizität der Kategorie 2 („Verdacht auf CMR-Potenzial für den Menschen“; carcinogen, mutagen, reproduktionstoxisch) vor. Die Einstufung von Ethanol in die Reproduktionstoxizitätskategorie 2 hätte zur Folge, dass der einzige Wirkstoff in Händedesinfektionsmitteln mit Wirksamkeit gegen unbehüllte Viren nicht mehr verfügbar wäre.

**Wissenschaftliche Bewertung der Unbedenklichkeit von Ethanol-basierten Händedesinfektionsmitteln (EBHD):**

Die verfügbaren epidemiologischen Studien bestätigen kein erhöhtes Krebsrisiko bei mit EBHD exponierten Menschen. Die vorliegenden Erkenntnisse deuten nicht darauf hin, dass der Wirkstoff beim Menschen wahrscheinlich Krebs verursacht, außer bei ungewöhnlichen oder unwahrscheinlichen Expositionswegen oder -höhen.

Die Evidenz für eine reproduktionstoxische Wirkung von Ethanol beruht auf dem Konsum von alkoholischen Getränken durch schwangere Frauen mit einer unvergleichlich höheren Ethanol-Aufnahme als durch Einsatz von EBHD. Die transdermale Ethanolaufnahme bei der Händedesinfektion ist bis zu zehnmal geringer als die orale Aufnahme von Getränken mit verstecktem Ethanolgehalt wie Apfelsaft, Kefir oder alkoholfreiem Bier. Der Blutspiegel liegt nach Händedesinfektion mit EBHD im physiologischen Bereich der Nahrungsaufnahme.

**Schlussfolgerung:**

Es gibt keine epidemiologischen Hinweise auf eine Toxizität für Arbeitnehmer beim Umgang mit ethanolhaltigen Produkten in der Industrie oder bei der Verwendung von EBHD in Gesundheitseinrichtungen. In Anbetracht der Tatsache, dass die Einstufung von EBHD in die Reproduktionstoxizitätskategorie 2 durch die aktuelle wissenschaftliche Forschung nicht bestätigt wird, und es keinen bioziden Ersatzwirkstoff zur Händedesinfektion mit Wirksamkeit gegen unbehüllte Viren gibt, empfehlen medizinische Fachverbände und Expertenausschüsse aus Europa, den USA, Kanada, der Asia-Pacific Region und die Weltgesellschaft für Virologie unmissverständlich und mit höchster Priorität, EBHD als Wirkstoff für PT1-Biozide zuzulassen und nicht in die Reproduktionstoxizitätskategorie 2 einzustufen

## Introduction

The biocidal products legislation in Europe is designed to reduce the number and quantity of chemicals used. However, ensuring hygiene in healthcare facilities is not a primary objective of this legislation, leading to an inherent conflict between the goal of minimizing chemical use and the necessity of employing disinfectants in sufficient variety and quantity to prevent infections both in healthcare settings and in the community [1]. Additionally, if an active agent – such as ethanol in hand rubs – faces a scientifically unjustifiable classification that prohibits its use, especially when no substitutes are available, this issue must be addressed with a sound scientific basis. Due to this critical situation, a comprehensive literature search was conducted to determine whether the use of ethanol for hand antisepsis poses any risk of reproductive toxicity [2]. The resulting memorandum [2] confirmed the safety of ethanol-based hand rubs (EBHR) for preventing infections in healthcare and community settings. It also stated that, among the active ingredients used in hand antiseptics, only ethanol is effective against non-enveloped viruses, unlike propanol and isopropanol [2].

## Legal and regulatory background

Alcohol-based hand rubs (ABHR) are classified as product type (PT) 1 under to Annex V of Regulation (EU) No 528/2012 (Biocidal Products Regulation) within the European Union and the European Economic Area [3]. This classification covers both hygienic hand rub and surgical hand preparation. According to the requirements of the Biocidal Products Regulation, active substances used in biocidal products must receive approval. Following this approval, all biocidal products must obtain authorization to be marketed. Unless they are authorized in accordance with Regulation No 528/2012 [2], biocidal products should neither be made available on the market nor used [4]. To assess efficacy during the authorization process, ECHA has issued guidelines that outline both general and specific requirements for individual product types, including those for the efficacy of hand rubs [1].

## The current classification of alcohol-based hand rubs (ABHR)

2-Propanol was approved as an active substance for use in biocidal products of PT 1 (human hygiene), PT 2 (disinfectants and algaecides not intended for direct application to humans or animals), and PT 4 (food and feed areas) through Implementing Regulation (EU) 2015/407 [5]. Similarly, 1-propanol was approved as an active substance for use in biocidal products of PT 1, 2, and 4 under Implementing Regulation (EU) 2017/2001 [6]. 

The classification of ethanol has remained unresolved since 2007 due to conflicting opinions among experts and authorities, leading to significant delays in its evaluation. Greece serves as the rapporteur Member State responsible for the evaluation of ethanol. Ethanol is also a candidate for substitution as an active substance under Article 10 of Regulation (EU) No 528/2012 in PT 1, 2, and 4 [4].

If deemed necessary ethanol should be classified as a carcinogen according to Regulation (EC) No 1272/2008 (CLP Regulation). According to ECHA, there is no general consensus among data submitters, although a minority (14.28% of REACH registrations) consider the substance as carcinogenic. Most of this minority suggests that the concern may be related to an impurity or additive rather than ethanol itself [1]. 

In the “Registry of intention” for classification and labeling, the Greek authority updated the harmonized classification and labeling of ethanol on 27 July 2020 [4]. The current proposal for extending this harmonized classification includes categorizing ethanol as a reproductive toxicity category 2, “suspected to have CMR potential for humans” (carcinogen, mutagen, reprotoxic). This represents a downgrade from the more severe classification of carcinogenic category 1A and reproductive toxicity 1A. However, it is important to note that the Risk Assessment Committee of the ECHA is not bound by the proposed classification. Therefore, the possibility that ECHA may still classify ethanol as carcinogenic and/or reproductive toxicity category 1 cannot be ruled out. Classifying ethanol as a CMR substance would result in the removal of the only active ingredient in hand antiseptics effective against non-enveloped viruses. The additional labelling required for a reprotoxic category 2 classification (e.g., “suspected of damaging the unborn child”, “may cause harm to breastfed children”, “avoid contact during pregnancy/while nursing”) would effectively amount to a de facto ban on ethanol-based hand rubs (EBHR). It is likely that many people would refuse to use EBHR due to such labelling. For example, the Pregnant Workers Directive could make it impossible to use these agents in healthcare settings.

Given the ongoing assessment of ethanol as a biocide, a memorandum was published in *Antimicrobial Resistance and Infection Control* in July 2022, by the Alcohol-Based Hand Rub Task Force, the WHO Collaborating Centre on Patient Safety, and the Commission for Hospital Hygiene and Infection Prevention at the Robert Koch Institute, Berlin, Germany [2]. This memorandum confirmed the safety of ABHR and their essential role in preventing infections caused by non-enveloped viruses.

## Synopsis of the toxicological evaluation of EBHRs

There is no epidemiological evidence of toxicity for workers from handling ethanol-containing products in industry or using EBHR in healthcare facilities [2]. 

The Poisindex^®^ [7] classifies ethanol as a category A3 carcinogen. This classification means that ethanol is carcinogenic in experimental animals at a relatively high doses, via specific route(s) of administration, at certain site(s), at histologic type(s), or mechanism(s) that may not be relevant to worker exposure. Available epidemiologic studies do not confirm an increased risk of cancer in exposed humans. The evidence does not suggest that ethanol is likely to cause cancer in humans except under uncommon or unlikely routes or levels of exposure [7].

The evidence for ethanol’s reprotoxic effects primarily stems from the consumption of alcoholic beverages by pregnant women, where ethanol uptake is incomparably higher [8]. The amount of transdermal ethanol absorption during hand antisepsis is up to tenfold lower than the oral intake from beverages containing hidden ethanol, such as apple juice, kefir or non-alcoholic beer. Blood level after hand antisepsis with EBHR remains within the physiological range associated with food intake [2]. Furthermore, the concentration of the ethanol metabolite ethyl glucuronide, a marker of ethanol consumption, in urine is below any harmful or toxic levels [2]. Studies show that dermal and inhaled ethanol absorption from using EBHR alone results in mean urinary ethanol concentrations that are, on average, over 60 times lower than those from the permitted use of alcohol-containing drinks, food, or cosmetic products [2].

The approval of propan-1-ol and propan-2-ol as biocides by the ECHA, alongside the continued lack of authorization for ethanol, is perplexing given the distinct differences in the metabolically-mediated physiological blood levels. After using EBHR, the increase in blood alcohol levels above baseline was approximately 157-fold, whereas the increase after using ABHR containing 1-propanol and 2-propanol was more than 1,800-fold and 10,000-fold, respectively [2].

Neither animal studies nor epidemiological analyses, nor the risk assessment of absorbed ethanol from medically indicated hand rubs, indicate any risk of toxicity, carcinogenicity, mutagenicity, or reprotoxicity from the repeated use of ethanol-based hand rubs as hand antiseptics [2].

## Spectrum of activity and indications of EBHR

Alcohol-based hand rubs are effective against a broad spectrum of vegetative bacteria, yeasts, and molds. However, among the three alcohols commonly used in hand rubs (ethanol, 2-propanol, and 1-propanol), only ethanol-based hand rubs (EBHR), either in a concentration of 95% or with reduced ethanol content combined with synergistic additives, are effective within 30–60 seconds against non-enveloped viruses such as adenoviruses, polioviruses, human enteroviruses, human papillomaviruses, polyomaviruses, echoviruses, and coxsackieviruses, as demonstrated in quantitative suspension assays [2].

Virucidal hand antisepsis is essential for preventing cross-infection with non-enveloped hydrophilic viruses, particularly when combined with virucidal surface disinfection [2], [9] in the following situations:

- In hospitals and doctor's practices, to prevent cross-infections between infected and healthy patients via healthcare workers
- In nursing homes and kindergartens to prevent cross-infections between infected and healthy humans via nursing staff
- To contain localized outbreaks, such as those on cruise ships
- To curb outbreaks and pandemics with non-enveloped viruses in the community
- To protect food from contamination during the manufacturing process, with the following ranking of outbreak risk: norovirus, hepatitis A and B virus, enteroviruses, rotavirus, coxsackievirus, echovirus, parvovirus, adenovirus [10]. The FDA Model Food Code [11] recommends the use of ethanol-based hand rubs (EBHR) as an alternative to handwashing when heavy soiling is absent.

For example, norovirus is responsible for approximately 58% of foodborne illness cases of known etiology [12], leading to around 125 million cases annually [13]. Extrapolating published data from 5 to 33 European countries, the estimated burden of disease caused by non-enveloped viruses includes over 300 million cases for rhinoviruses, 73 million for rotaviruses, over 20 million for papillomavirus 6, over 2 million for coxsackieviruses, over 500,000 for enteroviruses, 232,000–452,000 for noroviruses, over 100,000 for echoviruses, 5,000 for adenovirus as the causative agent of keratoconjunctivitis epidemic, 6,000 for hepatitis A virus, and 4,500 for hepatitis B virus [14].

Non-enveloped viruses that are highly transmissible via hands and pose significant outbreak risks, particularly in the absence of vaccines, include norovirus, rotavirus, adenovirus, enterovirus, coxsackievirus, reovirus, hepatitis A virus, hepatitis E virus, rhinovirus, bocavirus, and aphthovirus. Additionally, vaccine-preventable non-enveloped viruses such as papillomavirus and wild poliovirus type 1 (WPV1) in regions like Afghanistan and Pakistan, are also transmitted via hands.

## Conclusions

There is no reason not to use ethanol as a biocidal active ingredient in ethanol-based hand rubs (EBHR) for healthcare settings, the food industry, and public areas to prevent infections caused by non-enveloped viruses. The classification of EBHR as a reprotoxin category 2 is not supported by current scientific research, and no alternative biocidal substance with proven efficacy against non-enveloped viruses exists. Therefore, key organizations such as the WHO Task Force on Alcohol-Based Hand Rubs, the WHO Collaborating Centre on Patient Safety, Infection Prevention & Control, and Antimicrobial Resistance, the Commission for Hospital Hygiene and Infection Prevention, Robert Koch Institute in Berlin, Germany, and the Association for Applied Hygiene in Germany strongly recommend retaining ethanol as a crucial component in hand rubs for healthcare, safe food production, and community use, particularly during outbreak situations. The absence of effective hand rubs against non-enveloped viruses such as norovirus, adenoviruses, and enteroviruses D68 and 71 (which are not vaccine-preventable) poses significant public health risks. Additionally, economic losses will arise not only from increased morbidity but also from the potential need to relocate ethanol production outside Europe for domestic use. Moreover, it is currently unknown whether controlling outbreaks and pandemics caused by new and emerging viruses might depend on ethanol as the only effective virucidal agent available for hand antisepsis.

Medical associations and expert committees with specialized knowledge in infection prevention and clinical virology from Europe, the USA, Canada, the Asia-Pacific region, and globally active societies unequivocally recommend that ethanol-based hand rubs be approved as an active substance for PT1-biocides and not be classified as a reproductive toxicant category 2:

- Asia-Pacific Society for Infection Control
- Australasian College for Infection Prevention and Control
- Austrian Society for Hospital Hygiene
- Austrian Society for Hygiene, Microbiology and Preventive Medicine
- Bulgarian Association for Prevention and Infection Control
- Dutch Society for Infection Control
- European Society for Clinical Virology
- European Society of Clinical Microbiology and Infectious Diseases
- European Committee on Infection Control
- Infectious Disease Society of Finland
- Finnish Society for the Study of Infectious Diseases
- French Society of Hospital Hygiene
- Société de Pathologie Infectieuse de Langue Française
- German Association for the Control of Virus Diseases
- German Society for Hygiene and Microbiology
- German Society for Infectiology
- German Society of General and Hospital Hygiene
- Paul-Ehrlich-Society for Infection Therapy
- German Society of Virology e.V.
- Central Committee on Biological Safety, Germany
- Healthcare Infection Society
- Helenic Society for Infection Control
- Norwegian Society of Infectious Diseases
- Society for Healthcare Epidemiology America
- Sociedad Española de Enfermedades Infecciosas y Microbiología Clínica
- Spanish Society for Prevention Medicine
- Swiss Society for Microbiology
- Turkish Disinfection, Antisepsis and Sterilization Association
- World Society for Virology

## Notes

### Representatives

- **Didier Pittet**: Former Director of the Infection Control Program and WHO Collaborating Centre on Patient Safety, Infection Prevention and Control and Antimicrobial Resistance, University of Geneva Hospitals and Faculty of Medicine, Chairman for Clean Hospitals, Geneva, Switzerland^1^
- **Axel Kramer**: Past President of the German Society of General and Hospital Hygiene, Berlin, Germany; on behalf of the Board of the Society^2^
- **Martin Exner**: President of the German Society of the General and Hospital Hygiene, Berlin, Germany; on behalf of the Board of the Society^2^
- **Constanze Wendt**: Chairwoman of the Commission for Hospital Hygiene and Infection Prevention (KRINKO) at the Robert Koch Institute Berlin, Germany^2^

^1^ Representative of the WHO Collaborating Centre of Patient Safety

^2^ Representatives of the Commission of Hospital Hygiene and Infection Prevention at the Robert Koch Institute Berlin

### Competing interests

The authors declare that they have no competing interests.

### Authors’ ORCIDs

- Axel Kramer: 0000-0003-4193-2149
- Didier Pittet: 0000-0002-3667-7131

### Funding 

None.
